# Tracking Lung Tumors in Orthogonal X-Rays

**DOI:** 10.1155/2013/650463

**Published:** 2013-08-06

**Authors:** Feng Li, Fatih Porikli

**Affiliations:** Mitsubishi Electric Research Laboratories, Cambridge, MA 02139, USA

## Abstract

This paper presents a computationally very efficient, robust, automatic tracking method that does not require any implanted fiducials for low-contrast tumors. First, it generates a set of motion hypotheses and computes corresponding feature vectors in local windows within orthogonal-axis X-ray images. Then, it fits a regression model that maps features to 3D tumor motions by minimizing geodesic distances on motion manifold. These hypotheses can be jointly generated in 3D to learn a single 3D regression model or in 2D through back projection to learn two 2D models separately. Tumor is tracked by applying regression to the consecutive image pairs while selecting optimal window size at every time. Evaluations are performed on orthogonal X-ray videos of 10 patients. Comparative experimental results demonstrate superior accuracy (~1 pixel average error) and robustness to varying imaging artifacts and noise at the same time.

## 1. Introduction

Tumor tracking is an essential component of image-guided radiation therapy (IGRT) systems for treating lung and abdominal area tumors, which move significantly due to respiration. With accurate tracking, large margins added to the planning target volumes can be eliminated, and thus, adverse effects of radiation on healthy tissue can be minimized. Duration of treatment sessions can be shortened considerably by tracking tumors and applying radiation continuously.

Conventional tracking approaches determine tumor position using internal and/or external surrogates. Often, multiple passive (metallic) [1] and active (electromagnetic) [2] internal fiducials are implanted around tumor to continuously monitor its motion in X-ray videos. However, surgical implantation of such invasive internal fiducials are harmful as they destroy healthy tissues, and patients have greater chance of developing pneumothorax during CT-guided fiducial placement. Besides, internal fiducials slide and relocate during the course of multiple treatment sessions causing uncertainty in their reference positions.

It is also possible to apply correspondence models between external markers (chest and abdominal area pointers) and internal fiducials to indirectly estimate tumor position in optical images [3, 4]. Yet, the correlation between external markers and tumor position may be violated easily as a result of complex respiratory biomechanics [5]. Alternatively, parametric models of motion patterns are used to track tumors [6]. Such methods, however, require manual labeling of surrogate regions and tumor positions for a long period of time in order to train models. A multiple template matching method for X-ray images is described in [7]. It should be noted that template matching may fail for low-contrast tumor regions where the image quality is low.

**Figure 1:**
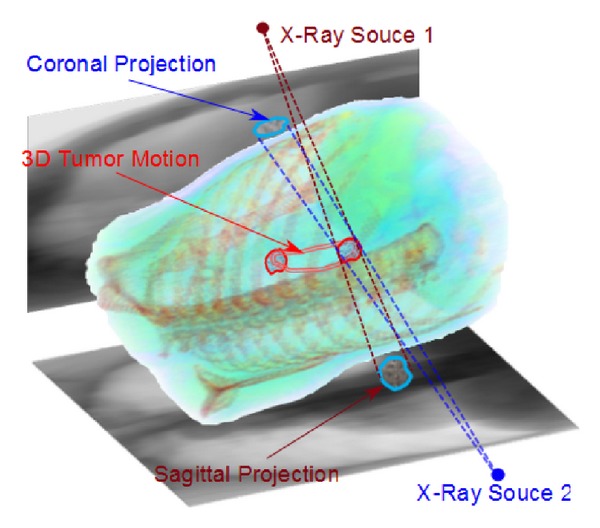
Orthogonal X-ray imaging. Due to breathing, projected regions in sagittal and coronal X-rays move significantly up to 3~4 cm.

 Here, we present a tumor tracking method that does not require any invasive internal fiducials or external markers. Unlike the traditional methods, we consider the tumor tracking problem as a regression model fitting task between orthogonal X-ray videos and underlying tumor motion as illustrated in Figure 1. Our intuition is that tumor motion in orthogonal planes can be approximated by affine motion (or a similar parametric motion) and image features can be linearly correlated with these tumor motion parameters. In other words, the feature vector we compute within the tumor region is supposed to be an indicator of the tumor motion. For this, we use an image feature that is sensitive to motion unlike the insensitive features such as intensity histograms that conventional motion estimation methods often require.

We learn an online regression model, which is a single matrix that maps image features to motion parameters using the initial pair of the orthogonal X-ray images. We randomly generate a set of 3D motion hypotheses (affine motion matrices) around the initial tumor location. To efficiently cover the parameter space, we generate motion hypotheses conditioned on the respiratory biomechanics (e.g., depending on the position of the tumor, the motion can be constrained to be translational only).

These hypotheses map an initial support window volume tumor in both orthogonal X-ray image pairs by simple 3D-2D projection. In addition, we determine an optimal tracking window size in each orthogonal view. We then compute two image feature vectors (e.g., histogram of oriented gradients) in the corresponding image windows, and we concatenate these vectors.

To learn the regression model, we solve an overcomplete least-squares fitting problem between the motion hypotheses and concatenated feature vectors using the geodesic distances. In [8, 9], a linear regression function is considered. Since affine motion matrices constitute a Riemmanian manifold, motion hypotheses distances should be computed through geodesics. The benefits of using Riemmanian manifold over previously used motion models in Euclidean space (e.g., just vectorizing the motion hypothesis matrix) can be found in [10] for nonmedical examples. We are inspired by [10], yet we significantly extend its basic idea to 3D tumor motion, multiple orthogonal videos, and joint feature computation while further refining it with an adaptive optimal window selection.

To estimate the current tumor position in a new X-ray pair, we simply apply the learned regression model to the concatenated feature vector computed in the new X-ray images within the previous tumor windows.

Note that we track the tumor position but not its boundary. Boundary tracking can be done by segmentation, registration, boundary fitting, B-splines, and so forth. However, for a low-contrast and invisible tumor, boundary tracking may not be possible since the tumor boundary is not distinct from its surrounding tissues. Our method can track the position of low-contrast tumors and does not require registration.

The presented regression method is possibly one of the fastest tumor tracking algorithms (and the fastest that we tested) as it only requires a feature vector and a matrix multiplication without any expensive search operation (template matching, etc.), iterative updates (mean shift), optimization with smoothing or total variation constraints (optical-flow), or testing a large number of hypotheses during the tracking process (particle filtering) as other tracking techniques. It does not require any offline training or patient-specific adaptation. It does not require a tumor detector either.

Extensive experiments with challenging real patient datasets demonstrate that the proposed method is robust to different tumor locations and very accurate for low-contrast tumors.

## 2. Regression Tracking

 Regression tracking learns an online regression model **Ω** to correlate the observed image features **X** to underlying tumor motion **Y**. This can be done either learning a single model in 3D Euclidean space (3DR) or learning two separate regression models in 2D planar space then intersecting them in 3D space (2DR).

We formulate the model learning as a fitting problem, which finds the regression coefficients **Ω** through minimizing the geodesic distance between the tumor motion matrices **Y** and its estimates **X** **Ω** as

(1) $$\begin{array}{c} \underset{\Omega }{\min}{||X\Omega -Y||}^{2}+\lambda {||\Omega ||}^{2}, \end{array}$$

where *λ* determines the weight of Tikhonov regularization, that is, preference to solutions with smaller smooth norms. We solve (1) through ridge regression to determine **Ω**. The solution is given as

(2) $$\begin{array}{c} \Omega ={\left(X^{⊤}X+\lambda I\right)}^{-1}X^{⊤}Y. \end{array}$$

At the first image pair (*t* = 0) of orthogonal X-ray sequences, we learn **Ω** using the initial location *M*_0_. Then, for any given image pair *t* > 0, we simply compute a feature vector *h*_*t*_ and apply **Ω** to find the tumor position:

(3) $$\begin{array}{c} M_{t}=M_{t-1}\cdot \exp\left(h_{t}^{⊤}\Omega \right). \end{array}$$

**Figure 2:**
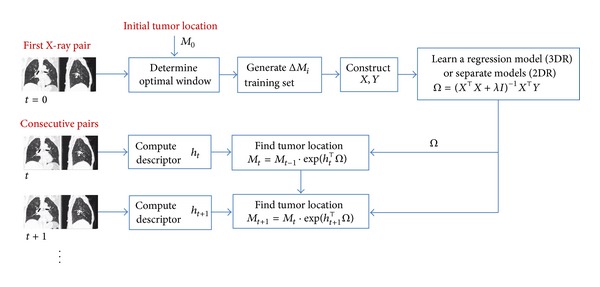
Learning of regression model and tracking tumor.

A flow diagram of our method is given in Figure 2. Next, we explain how to compute *h*_*t*_, **X**, and **Y** and details of our 3DR/2DR formulations.

### 2.1. Training

For a single X-ray sequence, the tumor motion between two consecutive frames can be modeled as a 3 × 3 affine matrix *M* (represented by 6 independent motion parameters in 9 coefficients). We use affine motion (translation, rotation, scale change, and skew) to represent the incremental movement of the tumor. Considering the biomechanical tissue models and respiratory mechanics, such an affine model is an adequate model. [11] further states that the tumor motion can be modeled as a simpler periodic function, featuring more time spent at the exhale phrase. When there is no hysteresis with the patient's respiratory system, the pathways for inspiration and expiration of one respiratory cycle are approximately the same and almost linear. Even with the presence of hysteresis, the maximum deviation between the two pathways is usually much smaller than the distance along the primary direction of the tumor motion. Thus, a simple translation only motion would be sufficient in most cases; however, we consider full affine motion in our analysis for the completeness of the discussion.

Let *M* represent the transformation from a unit square in the object space to the affine region enclosing the target tumor in the image space; that is, (*x*_img_,*y*_img_,1)^**⊤**^ = *M*(*x*_obj_,*y*_obj_,1)^**⊤**^. The tumor position *M*_*t*_ at time *t* can be computed efficiently from *M*_*t*−1_ by

(4) $$\begin{array}{c} M_{t}=M_{t-1}\cdot \Delta M_{t}, \end{array}$$

where Δ*M*_*t*_ is the incremental motion.

Affine motion matrices lie on a Riemmanian manifold. This means that we cannot simply vectorize the motion matrices to compute an Euclidean distance between them. Geodesic distances should be measured, thus we apply Lie group exponential map to determine Δ*M*_*t*_

(5) $$\begin{array}{c} \Delta M_{t}=\exp\left(h_{t}^{⊤}\Omega \right), \end{array}$$

where *h*_*t*_ ∈ ℝ^*m*^ is a *m* × 1 feature vector corresponding to the unit square in object space warped from *I*_*t*_ through *M*_*t*−1_, **Ω** is a *m* × *d* matrix of regression coefficients, and *d* is the number of motion coefficients. For 2D (3D) affine transformation *d* can be set to 6 (12) independent parameters, or all 9 (16) coefficients of the 3 × 3 (4 × 4) affine motion matrix. In either case, each row of **Ω** should be reshuffled to obtain the corresponding motion matrix.

**Figure 3:**
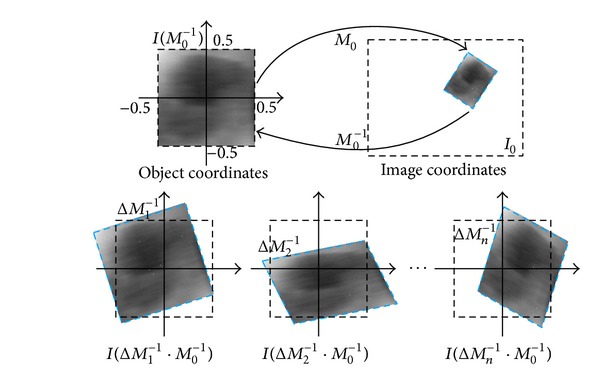
Affine transformation *M*_0_ maps a unit square from object space to image space. We generate *n* training hypotheses Δ*M*_*i*_^−1^ in object space and compute corresponding features in image space.

To find the optimal solution of (1), we first generate a training set of *n* random affine transformation hypotheses Δ*M*_*i*_ together with their corresponding feature vectors *h*_0,*i*_ extracted from image *I*_0_, as shown in Figure 3. Then, we construct **X** and **Y** as

(6) X=(h0,1⊤;…;h0,n⊤)n×m,Y=((log⁡ΔM1)⊤;…;(log⁡ΔMn)⊤)n×d.

Keep in mind that we generate more hypotheses than the feature vector size and significantly more than the number of motion parameters. This means that we obtain an overcomplete system of equations in above minimization. Random sampling serves to achieve an effective and reliable training set and avoid overfitting. One can also quantize the motion space and use those quantized motion parameter values in training. We observed that the value of *n* has little impact on the performance as long as it is larger than the feature vector dimension; that is, *n* > *m*. In our experiments, we set *n* = 600 for the 2D regression algorithm.

 Since the incremental motion of the tumor is small [11], we can also limit the sampling bounds using the maximum translational and rotational motion constraints typical for tumor motion. This not only improves the tracking accuracy but also stabilizes the tumor trajectories by removing possible jitters.

We use Histograms of Oriented Gradients (HOG) to describe the tumor window features *h*_*t*_. HOG has 8-bin histograms for each 5 × 5 block within the tracking window, concatenated into a single column vector.

During the tracking process, the model **Ω** can be relearned to adapt changes if necessary. In our simulations we have not observed any drift or model distortion issue with only one training at the initialization.

Note that the regression model **Ω** built using the first images of the orthogonal sequences where the initial tumor position is given (by table alignment, etc). Then, it automatically tracks the tumor in the newly given images. Our learning method is blind to patient data; it does not make any patient-specific assumption or require any patient-specific information. It does not use any offline training and does not require manual marking of the tumor trajectory either.

For 512 × 512 image resolution, automatic generation of motion hypotheses, computing the corresponding concatenated feature vectors and solving ridge regression, takes 0.05 seconds.

### 2.2. 3D Regression (3DR)

To learn a joint regression model that correlates the 3D tumor motion directly with the orthogonal X-ray sequences, we group the feature vectors from the two views and estimate a 3D affine motion corresponding to them directly. We construct the combined feature vectors (*h*_0,*i*_^1^; *h*_0,*i*_^2^) from the two X-ray views *I*_0_^1^ and *I*_0_^2^ based on the labeled tumor position at time *t* = 0 and randomly generate *n* 3D affine matrices Δ*M*_*i*_^⋆^ in 3D Euclidean space. Next, we project Δ*M*_*i*_^⋆^ on the orthogonal X-ray image planes. Within the corresponding regions, we extract the features *h*_0,*i*_^1^ and *h*_0,*i*_^2^ for the first and second X-ray views. In this case, **Ω** ^⋆^ maps combined feature vectors (*h*_*t*,*i*_^1^; *h*_*t*,*i*_^2^)  (*t* = 0, *i* = 1,…, *n*) to their corresponding 3D affine motion matrices Δ*M*_*i*_^⋆^, where Δ*M*_*i*_^⋆^ is a 4 × 4 affine matrix. Thus, **X** ^⋆^ = ((*h*_0,1_^1^;*h*_0,1_^2^)^**⊤**^; …; (*h*_0,*n*_^1^;*h*_0,*n*_^2^)^**⊤**^), **Y** ^⋆^ = ((log⁡Δ*M*_1_^⋆^)^**⊤**^; …; (log⁡Δ*M*_*n*_^⋆^)^**⊤**^).

### 2.3. 2D Regression (2DR)

Instead of learning a 3D regression model, we could simply learn two separate regression matrices **Ω**_1_ and **Ω**_2_ for the two X-ray views, and we apply (4) and (5) iteratively to track the tumor in each X-ray view. Once we have the two individual tracking results *p*_*t*_^1^ and *p*_*t*_^2^ of the two orthogonal X-ray views at time *t*, we can simply compute the tumor position *p*_*t*_ in 3D through back projection. To do this, we first connect *p*_*t*_^1^ and *c*_1_ to form lines *l*_*t*_^1^,  *p*_*t*_^2^, and *c*_2_ to form line *l*_*t*_^2^ and then compute *p*_*t*_ as the intersection of *l*_*t*_^1^ and *l*_*t*_^2^, while *c*_1_ and *c*_2_ are the source points of the two orthogonal X-ray radiation. In practice, *l*_*t*_^1^ and *l*_*t*_^2^ may not necessarily intersect with each other; thus we choose the midpoint of the shortest path connecting these lines to represent the tumor positions.

### 2.4. Optimal Tracking Window

Tracking window size plays an important role in tumor tracking in soft tissues, especially in lung and abdomen areas. To yield valid tracking results for template based methods, the tracking window should not be too big (may underestimate motion) or too small (may lose track). We examine the self-similarity in the local tumor region to find the optimal tracking window size for each patient data.

Given a candidate window size *w*_*x*_ × *w*_*y*_, we define the local search region of size 2*w*_*x*_ × 2*w*_*y*_ with the same center, and *r* = (*w*_*x*_ × *x*_*y*_)(*w*_*x*_^⋆^×*w*_*y*_^⋆^)^−1^ ∈ [0.5^2^, 1.5^2^], where *w*_*x*_^⋆^ × *w*_*y*_^⋆^ is the bounding size of the tumor. We compute the feature distance for any pair of image patches within the searching region using *ℓ* ^2^ norm and use the mean error as an indicator to describe the discriminatory power of the candidate window size.

Intuitively, the larger this mean error the more discriminatory power the candidate size has. However, there may exist different distance distributions, which have the same mean error. To take into account these cases, we give preference to the small (20% of) feature distances and use their mean as the indicator. In our experiments, the ratio *r* of the optimal windows size to the tumor bounding box varies from 0.9 to 1.2 for the coronal view and from 1.1 to 1.3 for the sagittal view for different X-ray data sets.

## 3. Results

 For objective performance evaluations, we test our tracking algorithm on digitally reconstructed radiograph (DRR) sequences obtained from real-patient 4DCT data. These DRR sequences have manually labeled ground-truth 3D landmark positions.

Using X-ray videos has several issues. To annotate ground-truth motion, X-ray videos should depict tissues with embedded metallic marker. However, it is problematic to make a tracking algorithm to ignore high contrast marker regions, which are often close to tumor and yet compute uncontaminated image features for an unbiased evaluation. Besides, markers themselves introduce uncertainty on the ground-truth data since they may dislocate from the initial calibrated positions or occlude each other in X-ray videos.

 We use orthogonal DRR sequences obtained from 10 patients' 4DCT data [12]. This data has different tumor locations, shapes, and internal volume characteristics.

**Figure 4:**
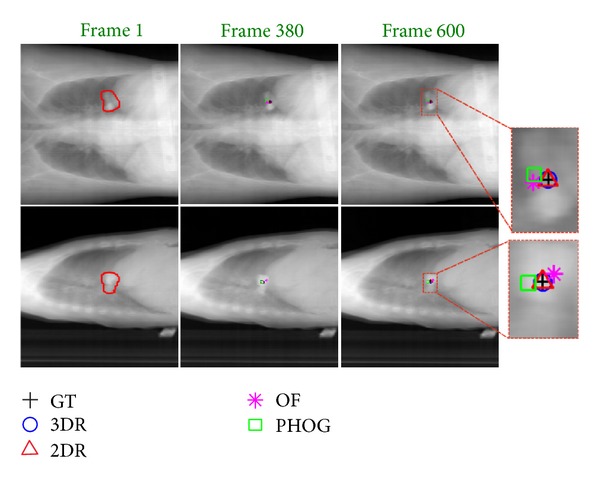
Orthogonal X-ray images from Patient 7. Frame 1 depicts the overlaid tumor boundary, and Frames 380 and 600 show the exhale phase and the inhale phase of the lung, respectively. We compare the performance of 3DR, 2DR, OF, and PHOG with the ground truth (GT) tumor center positions at Frames 380 and 600 (best in color).

Using a state-of-the-art simulator [13], we embed low-contrast tumors in different shapes, sizes, and locations in the original 4DCT data and then generate DRR sequences representing different breathing patterns. Tumor shapes range from spheroids to very intricate 3D polytopes. Each test case is tested with a different regular breathing signal and two irregular breathing patterns. Since we do not impose any temporal smoothing or linear dynamical model (Kalman filter), the performance is not affected by the different breathing patterns. Each DRR sequence we test has around 900 frames.  Figure 4 shows sample coronal and sagittal views.

Compared with higher resolution flat-panel X-ray digitizer, this data presents considerable challenges.

- Since the patient CT has limited number of slices, it is low resolution and drastically blurred particularly in the cranial-caudal direction of the coronal view; for example, ~100 × 256 pixels (underlying DRR are of 256 × 256) for Patients 1–5 and ~128 × 512 (DRR 512 × 512) for Patients 6–10. On the other hand, a typical of the flat panel X-ray digitizer has 2048 × 2048 pixels. Limited resolution causes less discriminative features leading potential tracking failures.
- It has low contrast. The typical dynamic range of a commercial flat-panel digitizer is 16 bits. However, the DRRs we use are 8 bits encoded to push the algorithm to its limit.
- It contains noise and imaging artifacts. Unlike X-ray videos from digitizer, DRR sequences inherit all 4DCT imaging artifacts due to limited CT scanning speed.
- We add white random Gaussian noise to the DRR sequences considering that DRR images might not suffer from X-ray image acquisition noise.

These issues certainly make tumor tracking more difficult in our dataset.

We compare the performance of our 3DR and 2DR methods with the state-of-the-art including the best existing optical flow implementation (OF) [14] and *ℓ*_1_-based particle filter (P*ℓ*_1_) [15]. [14] combines the “classical” flow formulation with image boundaries and designs an optimization framework that utilizes median filtering for flow field estimation. [15] is a template-based robust visual tracking method, which enforces sparse representation on the template set and follows a particle filter-based Bayesian state inference.

We also implemented a HOG based particle filter (PHOG) algorithm, which uses the same number of 3D particles as the P*ℓ*_1_ method but computes the observation likelihood from HOG feature matching. The OF algorithm first finds the tumor motions on the two X-ray views and then estimates the 3D tumor position through back projection. We compute the optical flow between two consecutive frames in full resolution since that the magnitude of the tumor motion is unknown and far away feature points can also contribute to the motion estimation of tumor regions.

Table 1 presents the detailed performance comparison of these algorithms as well as their average processing time per frame. For each test case, we also list the tumor motion magnitude in the last column. We use the Euclidean distance between the estimated and the ground truth (GT) tumor center in 3D as the error measurement (in pixels). The GT tumor center is calculated as the mass center of the GT tumor, while the estimated tumor center is the center of the estimated tracking window.

Our results using 10 different patient data show that 2DR gives 1.05 pixel, 3DR 1.16 pixel, OF 3.57 pixel, P*ℓ*_1_ 5.01, and PHOG 5.68 pixel error on average where the average tumor displacement in the GT is 13.86 pixels. This means that 2DR estimates are 92.5% accurate (1.05 is 7.5% of 13.86).

From Table 1, we can see that 3DR and 2DR consistently achieve most accurate tracking results. Other algorithms vary significantly for different test cases and may lose track of the tumor under certain scenarios (e.g., for Patient 10).

We do not claim that just because we achieve a 1.05 pixel average error on lower-resolution images we may obtain the same error at higher resolutions. Our experimental results on the 256 × 256 and 512 × 512 datasets clearly show that the estimation errors in terms of *pixels* remains the same (in fact becomes lower: from 1.63 pixel average error for 256 × 256 to 0.69 pixel average error for 512 × 512) when the image resolution increases.

In Table 1, 4DCT data for Patients 1–5 have 256 × 256 DRR image size, while Patients 6–10 have 512 × 512. Even though the underlying total displacement doubled from 8.49 pixel to 19.23 pixel on average (implying the tracking problem becomes more challenging), the tracking errors in terms of *pixels* are better for 512 × 512 sequences. In other words, we can confidently expect similar pixel errors when we use even higher resolution images (as a result, get lower mm error). Instead of DRR, if we used a commercial product (e.g., Siemens Axiom Luminos dRF flat detector), 1.05 pixel error would correspond to 0.14 mm to 0.42 mm error.

Here, we also like to mention that, for a fair assessment of tracking methods, it is essential to report pixel errors rather than mm errors that is commonly conveyed in medical literature due to bottom-line clinical requirements. However, by measuring mm error, the same algorithm can produce different mm errors using different resolution input data. This does not mean that the algorithm gets any better or worse as nothing algorithmically changes.

**Figure 5:**
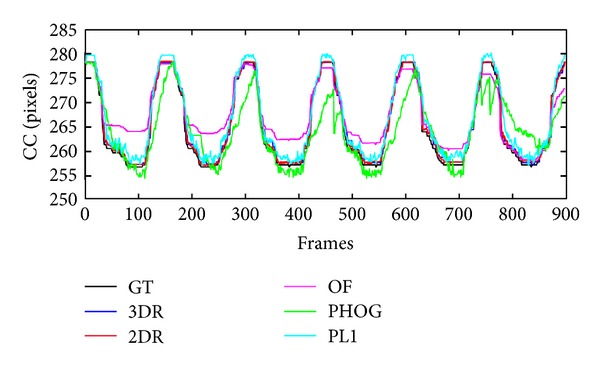
Estimated tumor trajectories of different tracking algorithms are compared with the ground truth data in the Cranial-Caudal (CC) direction for Patient 7. Note that P*ℓ*_1_ has large tracking errors in the axial plane.

In Figure 4, we show the tracking results of different algorithms on data set Patient 7, and our 3DR/2DR tracks the tumor very well for both inhale and exhale phases. In Figure 5, we draw the estimated tumor positions in the Cranial-Caudal (CC) direction along with the ground truth for Patient 7. It is apparent that the optical flow based tracker tends to underestimate the tumor motion causing significant errors when the tumor motion is large. The particle filter based algorithms (P*ℓ*_1_ and PHOG), on the other hand, exaggerates the tumor motions at the two extremities and produces jittery results. Another issue with the particle filter based trackers is that the lack of sufficient texture in X-ray images sometimes causes the selection of wrong motion hypothesis as the mode of the approximated pdf after the importance sampling.

Computationally optical flow based tracker (OF) is the slowest one among all trackers taking about 2.8 minutes to process a single frame on an Intel 3.4 GHz CPU, which is prohibitive for real-time tasks. There are of course faster OF methods, but their accuracy is worse. Our method can track the tumor in real time: 3DR (2DR) in less than 0.03 (0.06) seconds. 3DR algorithm can be preferred over 2DR for two reasons. First, 3DR generates tracking results twice faster than 2DR. Second, 3DR can avoid the divergence between two orthogonal views by learning a joint 3D regression model and maintaining the tumor positions in 3D space as opposed to two 2D planes.

Note that we do not train and test on the same images. We train on the initial pair where the tumor position is known *and then* track on the rest of the sequence automatically. Since the training step takes less than 0.05 seconds, this algorithm can run under any real-time clinical setting.

## 4. Conclusion

 We presented a noninvasive tumor tracking method and demonstrated that this tracker outperforms the state-of-the-art both in accuracy (~1 pixel error) and speed (0.03 sec). This corresponds to 7.5% tumor positioning error with respect to maximum tumor dislocation. Such a small tumor location error significantly reduces the prescribed treatment volume margins from several centimeters to millimeter range, and thus, prevents radiating healthy tissue in IGRT systems [16]. As a future study, we will extend the regression model to incorporate biomechanical tissue constraints for very complex tumor shapes.

## Figures and Tables

**Table 1:** Performance comparison (in pixel) of different tracking algorithms. 3DR and 2DR are very robust and consistently achieve most accurate tracking results (the best tracking result is in bold).

	3DR	2DR	OF	PHOG	P*ℓ*_1_	Total displacement
	~0.03 secs	~0.06 secs	~2.8 mins	~3.2 secs	~34.4 secs	
Patient 1	0.78 ± 0.34	0.52 ± 0.29	2.15 ± 0.79	1.63 ± 0.36	3.28 ± 1.30	8.03
Patient 2	2.61 ± 1.62	2.27 ± 1.37	2.12 ± 0.90	1.88 ± 0.96	7.54 ± 3.99	7.76
Patient 3	0.83 ± 0.42	0.70 ± 0.38	1.10 ± 0.43	2.06 ± 2.11	3.39 ± 0.83	10.47
Patient 4	2.86 ± 1.48	2.67 ± 1.41	4.20 ± 1.10	2.54 ± 0.84	4.38 ± 1.59	10.98
Patient 5	1.07 ± 0.54	0.92 ± 0.50	2.16 ± 0.90	2.14 ± 1.28	4.36 ± 1.19	5.23
Patient 6	0.65 ± 0.54	0.67 ± 0.48	10.98 ± 3.40	2.45 ± 1.50	5.76 ± 1.18	18.31
Patient 7	0.99 ± 0.49	0.94 ± 0.45	4.80 ± 1.87	4.71 ± 2.80	5.94 ± 1.49	22.13
Patient 8	0.69 ± 0.37	0.72 ± 0.39	3.15 ± 1.29	7.45 ± 8.30	5.19 ± 1.22	22.36
Patient 9	0.33 ± 0.29	0.35 ± 0.27	1.20 ± 0.60	3.07 ± 3.27	3.09 ± 1.01	7.28
Patient 10	0.82 ± 0.46	0.77 ± 0.45	3.92 ± 1.80	28.94 ± 11.52	7.16 ± 3.14	26.07
